# The whole-genome sequence analysis of *Morchella sextelata*

**DOI:** 10.1038/s41598-019-51831-4

**Published:** 2019-10-25

**Authors:** Mei-Han, Qingshan-Wang, Baiyintala, Wuhanqimuge

**Affiliations:** 1 Inner Mongolia ZhongXing Agriculture and Animal Husbandry Development Co., Ltd., Ulanqab, China; 2 Inner Mongolia Mang Lai Food Co., Ltd., Hohhot, China; 3 Innovative Mongolian Pharmaceutical Preparations Laboratory of Inner Mongolia, Inner Mongolia International Mongolian Hospital, Hohhot, China

**Keywords:** Genome-wide association studies, Genome duplication

## Abstract

*Morchella* are macrofungi and are also called morels, as they exhibit a morel-like upper cap structure. Morels contain abundant essential amino acids, vitamins and biologically active compounds, which provide substantial health benefits. Approximately 80 species of *Morchella* have been reported, and even more species have been isolated. However, the lack of wild *Morchella* resources and the difficulties associated with culturing *Morchella* have caused a shortage in the morels available for daily consumption. Additionally, in-depth genomic and morphological studies are still needed. In this study, to provide genomic data for further investigations of culturing techniques and the biological functions of *Morchella sextelata (M. sextelata)*, de novo genome sequencing was carried out on the Illumina HiSeq. 4000 platform using both the Illumina 150 and PacBio systems. The final estimated genome size of *M. sextelata* was 52.93 Mb, containing 59 contigs and a GC content of 47.37%. A total of 9,550 protein-coding genes were annotated. In addition, the repeat sequences, gene components and gene functions were analyzed using various databases. Furthermore, the secondary metabolite gene clusters and the predicted structures of their products were analyzed. Finally, a genomic comparison of different species of *Morchella* was performed.

## Introduction

*Morchella* is a member of the Morchellaceae family in the Pezizales order of the Pezizomycetes class under the Ascomycota division in the fungi kingdom. *Morchella* has a beautiful cap with a honeycomb-like structure and a brown, yellow, black or pale color that looks similar to lamb tripe, giving it the name “morel”. *Morchella* has been reported to have a high edible value because it contains sufficient essential amino acids, vitamins, mineral elements and proteins^1,2^.

*Morchella* was identified as early as 1882 and has rapidly expanded in recent years, not only because of its rich nutritional value but also because of its diverse biological functions, such as anti-inflammatory, anti-oxidative, antiviral, and antitumor effects^3–5^. Moreover, *Morchella* is also a great source of biomacromolecules, including polynucleotides, polysaccharides, proteins and small molecules^4^. Some metabolites prepared from *Morchella* have been used in tumor treatment^4^. Furthermore, some extracts from *Morchella* have been useful and helpful in the therapies of cancers, diabetes, and cardiovascular diseases^6^. Therefore, in recent years, *Morchella* has become more popular. However, naturally growing *Morchella* is very rare and expensive and thus insufficient to meet the demands of consumers^7^.

In the latest index of fungorum at http://www.indexfungorum.org/names/names.asp, 334 isolates of *Morchella*, including subspecies, have been reported. In the literature, approximately 80 species of *Morchella* have been identified since the beginning of this century^7^, and even more species have been discovered. Several species, *M. rufobrunnea*, *M. sextelata*, *M. eximia* and *M. importuna*, have been successfully cultivated in China^8^. The research on *Morchella* has increased dramatically in recent years and started to focus on the techniques of artificial planting and the biological functions of *Morchella*. However, there is still a lack of information about their genome to improve culturing techniques and to screen good species.

The genome sequence of *M. conica* (48.2133 Mb), *M. importuna* (median total length of 2 assemblies: 49.967 Mb), *M. septimelata* (median total length of 3 assemblies: 49.9613 Mb), and *M. eximia* (total length 73.461 Mb) have recently been reported in NCBI GenBank. However, genetic information about *M. sextelata* has not yet been reported.

The *M. sextelata* isolate studied here was successfully cultivated in Inner Mongolia, China, using relatively simple techniques and low-cost materials. Interestingly, the *M. sextelata* isolate cultivated here was larger, and its output was higher than that of other morels cultivated in other areas (Fig. S1). To investigate the genetic information and provide the data for further study of the biological functions of *M. sextelata*, a de novo sequence analysis was conducted, and its genome was assembled. Additionally, the protein-coding genes, gene components, related biological functions, and secondary metabolite gene clusters were analyzed. Finally, the gene expression in several species of *Morchella* was compared in this study.

## Results

### DNA extraction and quality test

Approximately 16.57 µg of DNA was extracted from the fresh *Morchella* strain. The quality test results showed that the purity (A260/A280) of DNA from the sample was 1.87, and the band of DNA on the gel after electrophoresis was clear, indicating that there was no protein, genomic, and other contamination. Thus, the DNA sample from the *Morchella* sample was sufficient for further analyses (Fig. S2). Before starting further analyses, the internal transcribed spacer (ITS) of DNA was sequenced, and ITS identification was performed by comparing the sequence to the sequences of known fungi in NCBI GenBank. Consequently, the *Morchella* strain was identified and confirmed as *M. sextelata*.

### Second-generation genome sequencing and assembly

Illumina PE150 sequencing generated 2,985 Mb raw reads, and 2,600 Mb clean reads were retained after the quality-control steps (data not shown). Then, the *M. sextelata* genome size was estimated to be 58.77 Mb after revision by K-mer spectrum analysis with K-mer = 15 and depth 27.36. The heterozygous rate was 0.10%, and the repeat rate was 36.09% (Table S1).

The clean reads were de novo assembled, and their scaffolds and contigs were analyzed. Notably, the generated results were only used to provide a reference for the following PacBio RSII third-generation analysis. Therefore, not all data are shown.

### Third-generation genome sequencing and assembly

**Figure 1:**
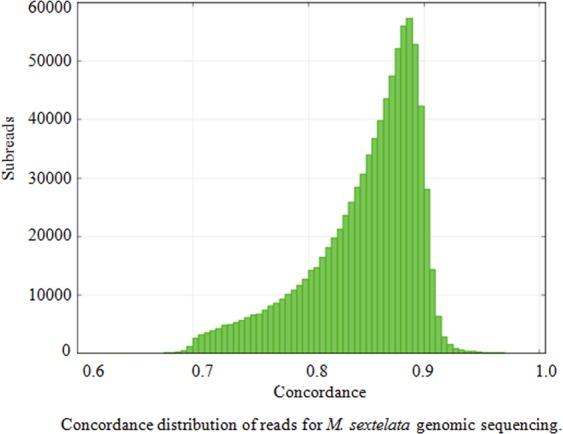
Horizontal coordinates represent the sequencing quality. The bars correspond to the left vertical coordinate, which shows the reads relevant to the quality of each sequencing data set.

**Figure 2:**
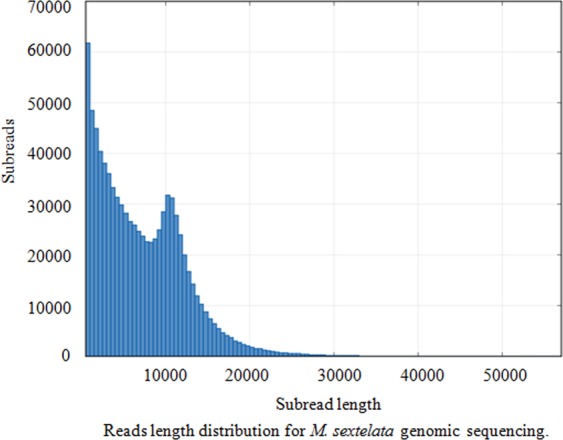
Horizontal coordinates represent sequencing read lengths. The bars correspond to the left vertical coordinate, which shows the read number relevant to each read length.

The raw reads from PacBio RSII were filtered by removing the reads with low quantity, and 869,182 clean reads were ultimately obtained (Table S2). The mean concordance of reads was 0.84 (Fig. 1), the mean read length was 6,626 bp and the N50 read length was 10,165 bp (Fig. 2).

The final clean reads from the third-generation PacBio RSII sequencing data were assembled, and a total of 59 contigs with N50 lengths of 1,569,782 bp were identified. The GC content of the whole-genome sequence was 47.37%. Finally, the whole genome was generated, and the estimated size was 52,925,331 bp (approximately 52.93 Mb) (Table 1). This was smaller than the initially predicted size of 58.77 Mb by the Illumina 150 system.

**Table 1:** The contig statistics of the assembled genome of *M. sextelata*.

Sample ID	Contig	Max Length (bp)	N50 Length (bp)	Total Length (bp)	Sequence GC (%)
*M. sextelata*	59	4,823,818	1,569,782	52,925,331	47.37

Note: N50 length indicates the length of the contig that localized to 50% of the total contig length when the contigs are arranged from longer to shorter sizes.

This Whole Genome Shotgun project has been deposited at DDBJ/ENA/GenBank under the accession SDUU00000000. The version described in this paper is version SDUU01000000.

### Genome annotations

**Figure 3:**
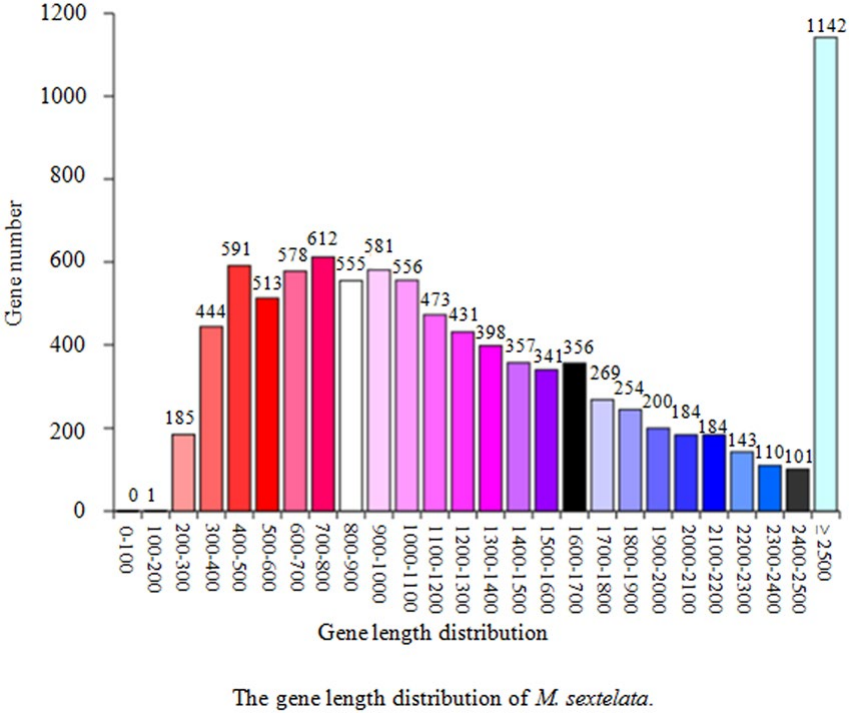
The horizontal coordinate represents the length of protein-coding genes, and the vertical coordinate shows the number of protein-coding genes. The number written on the top of the bar is the number of genes within each range of gene lengths.

The protein-coding genes in the genome of *M. sextelata* were de novo predicted using the Augustus 2.7 program. A total of 9,550 protein-coding genes with a total length of 13,107,305 bp, accounting for 24.77% of the whole genome (52,925,331 bp), were predicted. The lengths of the protein-coding genes mostly ranged from 300 to 1,700 bp and were 1,372 bp on average. The internal gene length was 39,818,026 bp, which accounted for 75.23% of the whole genome (Fig. 3) (Table 2).

**Table 2:** The summary of the final genome size and protein-coding gene annotation.

*M. sextelata*	Number or content
Genome size (bp)	52,925,331
Gene number	9,550
Gene total length (bp)	13,107,305
GC content in genes (%)	53.53
Gene content in genome (%)	24.77
Gene average length (bp)	1,372
Internal gene length (bp)	39,818,026
Internal gene GC content (%)	45.34
Internal gene content in genome (%)	75.23

### Genome component predictions

The interspersed repetitive sequences (IRSs) and the tandem repeats (TRs) were determined. IRSs contain short interspersed nuclear elements (SINEs) and long interspersed nuclear elements (LINEs), with the latter often showing transposition activity. In *M. sextelata*, a total of 15,485 TRs with repeat sizes of 1-1,894 bp, 10,026 minisatellite DNAs with repeat sizes of 10–60 bp and 2,702 microsatellite DNAs with repeat sizes of 2–6 bp were predicted (Table S3).

Noncoding RNAs (ncRNAs) play essential roles in biological processes, although they do not transcribe proteins. The results showed that there were 445 tRNAs with an average length of 85 bp in the *M. sextelata* genome. Compared to the amount of tRNA, the amounts of rRNA and snRNA were much lower, only 55 and 30, respectively. However, no rRNAs, sRNAs or miRNAs were predicted (Table S4).

### Gene function

The functions of genes were predicted by different databases. The number of genes enriched in each functional classification is shown in Table 3. Generally, many more coding genes were enriched in Kyoto encyclopedia of genes and genomes (KEGG) and gene ontology (GO) compared to other databases.

**Table 3:** The summary of gene function annotation in different databases.

*M. sextelata*	Enriched gene number
NR	6009
Swiss-Prot	2702
KEGG	5839
KOG	2148
TCDB	324
GO	5876
PHI	810
DFVF	635
P450	61
Secretary protein	542
CAZy	290

*NR: non-redundant protein database, KOG: eukaryotic orthologous groups, TCDB: transporter classification database, PHI: pathogen host interactions, DFVF: database of fungal virulence Factors, CAZy: carbohydrate-active enzymes database.

In the GO analysis, a total of 5,876 genes were enriched. Among them, 2,184 genes were associated with cell, the same number of genes contributed to cell part, and 1,024 genes were enriched in organelle. A total of 3,381 genes were predicted to have binding abilities. The catalytic activity and transporter activity for 2,986 and 389 genes were predicted. Various biological processes were predicted to be mediated by the genes of *M. sextelata*. For instance, 3,335, 3,239, and 859 genes possibly participate in cellular process, metabolic process, and localization, respectively (Fig. S3).

KEGG pathway analysis was conducted, and the enrichment of genes in different processing, metabolism, and organismal systems was annotated as shown in Fig. S4. The results showed that 203 genes were enriched in the pathway of infectious disease, 110 and 91 genes were enriched in the signaling of cancers and neurodegenerative disease, and 56 genes were enriched in the process correlated with endocrine and metabolic disease.

The genes were also classified based on COG functional database. The majority of annotated genes (331) were predicted to have a general function only. The top three enriched categories were 1. posttranslational modification, protein turnover, chaperones (240 genes); 2. translation, ribosomal structure and biogenesis (219 genes); and 3. amino acid transport and metabolism (161 genes) (Fig. S5).

NR is a database established by NCBI for the annotation of a wide range of information, including species details. Here, most of the genes were annotated to be nonredundant proteins in *Tuber melanosporum*, 146 of the genes were annotated in *Podospora anserine*, and 84 genes were annotated in *Pseudogymnoascus pannorum*. The top 20 predicted nonredundant protein species are shown in Fig. S6.

**Figure 4:**
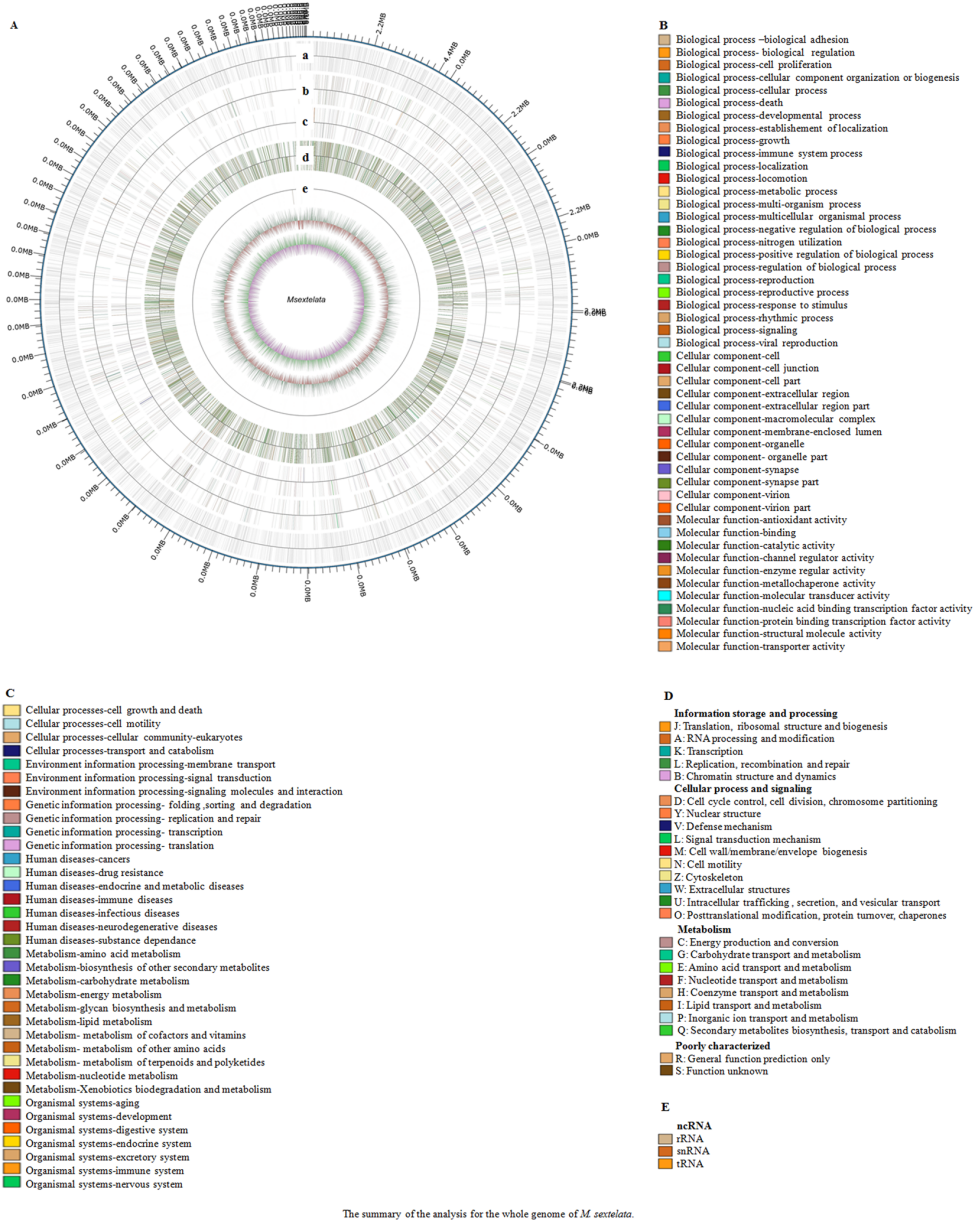
(**A**) The genetic map of *M. sextelata*. The outer ring is the gene position on the *M. sextelata* sequence. The ring (a) represents the information of coding gene positions. (b–d) represent the information of gene functions in the KOG, KEGG, and GO databases, respectively. (e) shows the ncRNA information. The outside ring is the positive chain, and the inner ring is the negative chain. The colors correspond to different functional characteristics predicted in the GO, KEGG, and KOG databases and different ncRNAs. (**B**) GO legend for (**A**). (**C**): KEGG legend for (**A**). (**D**) KOG legend for (**A**). (**E**) ncRNA legend for (**A**).

Based on the above gene annotation and function analysis, the genomic map was drawn and is shown in Fig. 4.

In addition, the function of proteins was classified using TCDB. As shown in Fig. S7, most transporter proteins were enriched in primary active transporters, up to 120. Next, 111 proteins were annotated as electrochemical potential-driven transporters, of which 55 were enriched in channels and pores.

There were 51 genes annotated in the endoplasmic reticulum retrieval protein1 (Rer1; putative heavy metal transporter) family, 30 genes in the major facilitator superfamily (MFS), and 28 genes in the H^+^- or Na^+^-translocating NADH dehydrogenase (NDH) family. Additionally, some genes were predicted to be members of transporter families, such as the genes A3238 and A0956 in the ammonia transporter channel (Amt) family, A2965 and A3464 in the CorA metal ion transporter (MIT) family, and A8387, A8696, A9300, A1158, A1940, A4798 in the drug/metabolite transporter (DMT) Superfamily.

The CAZy database includes glycoside hydrolases (GHs), glycosyltransferases (GTs), polysaccharide lyases (PLs), carbohydrate esterases (CEs), and auxiliary activities (AAs). When the genes of *M. sextelata* were annotated by the CAZy database, GHs were the most abundant enzymes, as 159 genes were predicted to be GHs. As shown in Table 4, 57 of the genes were predicted to code carbohydrate modules (CBMs), while 44 of the genes were estimated to be AAs. In addition, the functions of *M. sextelata* genomes were also annotated by the Pfam and Swiss-Prot databases (data not shown).

**Table 4:** Summary of Carbohydrate-Active Enzymes database annotation.

CAZy_class	Match number
CBM	57
CE	13
GH	159
GT	41
PL	20
AA	44

**Figure 5:**
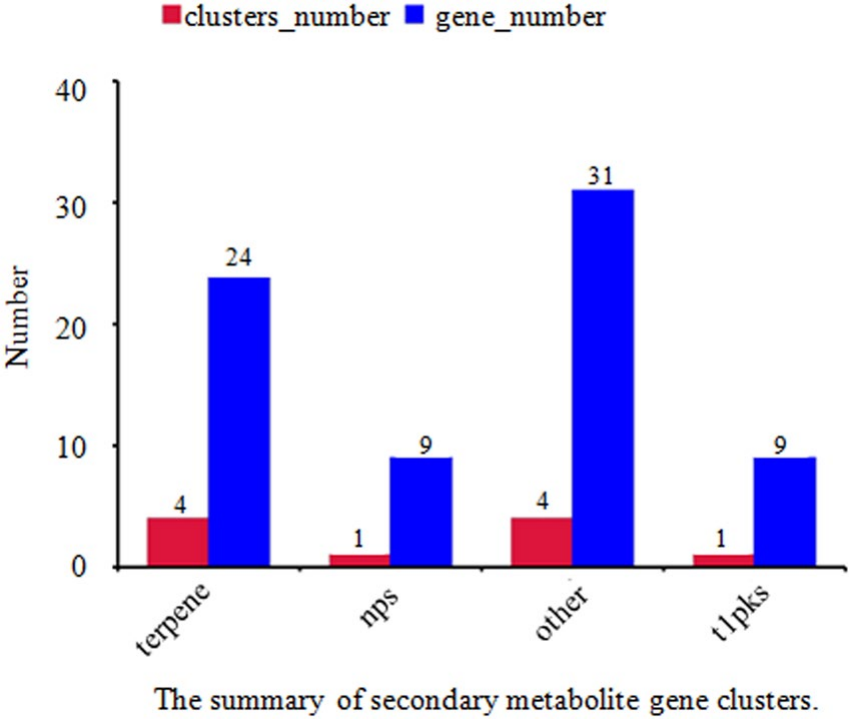
The horizontal coordinate represents the gene cluster name, and the vertical coordinate shows the number of gene clusters and genes in each cluster. Blue bars correspond to the gene number in each gene cluster, while red bars represent the number of clusters.

**Figure 6:**
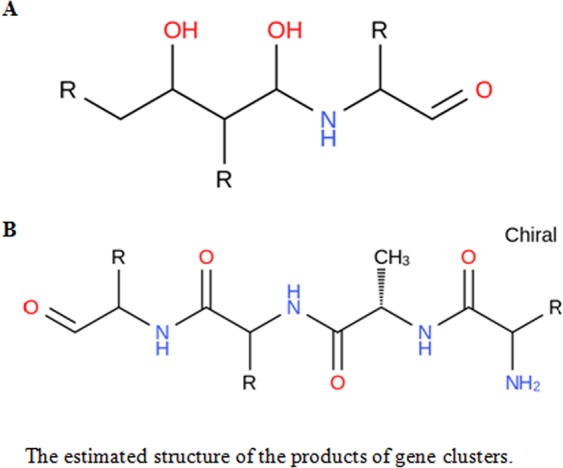
A: The predicted structure of the product of gene cluster 6; **B**: the predicted structure of the product of gene cluster 7.

The secondary metabolite gene clusters were analyzed. A total of 10 clusters, including 4 terpenes, 1 nps, 1 t1pks and 4 other clusters, were identified. The gene numbers for each cluster are marked on the blue bar (Fig. 5, Table 5 and Table 6). Additionally, the products of the gene clusters t1pks (polyketide synthase) and nrps (nonribosomal peptide synthase) were analyzed, and the chemical structures were predicted as shown in Fig. 6.

**Table 5:** The summary of secondary metabolite gene clusters.

Clusters	Cluster number	Gene number
terpene	4	24
nrps	1	9
t1pks	1	9
other	4	31

**Table 6:** The statistics of secondary metabolite gene clusters.

	Clusters	Contigs	Genes
1	terpene	Contig3	A6295; A6296; A6297; A6298
2	other	Contig4	A7647; A7648; A7650; A7651; A7652; A7653; A7654; A7655; A7656
3	other	Contig8	A9156; A9157; A9158; A9159; A9160; A9161; A9162; A9163
4	terpene	Contig11	A0451; A0452; A0453; A0454; A0455
5	terpene	Contig14	A1287; A1288; A1289; A1290; A1291; A1292; A1293; A1295; A1296; A1297
6	t1pks	Contig20	A3426; A3427; A3428; A3429; A3430; A3431; A3432; A3433; A3434
7	nrps	Contig27	A4555; A4556; A4557; A4558; A4559; A4560; A4561; A4562; A4563
8	other	Contig28	A4609; A4610; A4611; A4612; A4613; A4614; A4615; A4616
9	other	Contig33	A5824; A5825; A5826; A5827; A5828; A5829
10	terpene	Contig36	A6021; A6022; A6023; A6024; A6025

In the effector analysis, the cytochrome P450 database was BLAST searched to annotate the genes encoding fungal cytochromes. The results showed that 62 of the genes were predicted to be P450s distributed in 6 classes. Specific gene IDs are provided in Table 7.

**Table 7:** The statistics of P450 genes.

P450 class	Class name	Gene number	Gene
1	P450, CYP52	7	A7570, A0041, A7809, A2469, A1467, A3417, A4602
2	E-class P450, CYP2D	2	A7321, A1285
3	Undetermined	8	A8534, A8641, A1943, A1303, A0047, A0110, A1004, A1375
4	Cytochrome P450	11	A6397, A8907, A2892, A7709, A0184, A3635, A4884, A6462, A2432, A5836, A5236
5	E-class P450, group I	25	A2224, A0822, A0168, A1345, A7129, A6827, A6624, A9296, A7194, A0622, A2360, A3045, A8405, A6914, A1184, A1955, A9488, A5674, A9367, A9393, A4439, A2648, A8332, A5691, A0796
6	E-class P450, group IV	8	A1869, A6522, A6749, A0104, A0103, A3061, A4737, A5331

### Comparative genomic analysis

First, the genomic assembly results for *M. sextelata*, *M. septimelata*^9^, *M. eximia*^9^, *M. importuna*^3^, and *M. conica*^10^ were compared and are summarized in Table 8.

**Table 8:** Genomic assembly statistics of different species of *Morchella*.

	Species	Strain	Assembly (Mb)	GC%	Scaffolds	Contigs	N50	Genome coverage	INSDC	Sequencing	Assembly method
1	*Morchella septimelata*	MG91	49.62	47.1	3707	5121	28400	58x	QLOX00000000.1	Illumina	platanus version v. 1.2.1
2	*Morchella eximia*	MG90	73.46	46.0	7793	10613	26474	57x	QMFK00000000.1	Illumina	platanus version v. 1.2.1
3	*Morchella importuna*	M04 M26	51.08	47.3	106	110	958716	298x	QOKS00000000.1	Illumina HiSeq	AllPaths v. 44849
4	*Morchella conica*	CCBAS932	48.21	47.2	540	2145	52248	67.8x	PZQV00000000.1	Illumina	AllPathsLG v. R47710
5	*Morchella importuna*	M04 M24	48.86	47.0	323	504	362388	210x	QORM00000000.1	Illumina HiSeq	AllPaths v. 44849
6	*Morchella septimelata*	MG91	49.96	—	5231	5241	37765	151.17x	PYSJ00000000.1	Illumina GAIIx	SPAdes v. 3.0
7	*Morchella septimelata*	MG113	51.40	—	9172	11637	28426	88x	QMFJ00000000.1	Illumina	platanus version v. 1.2.1

**Figure 7:**
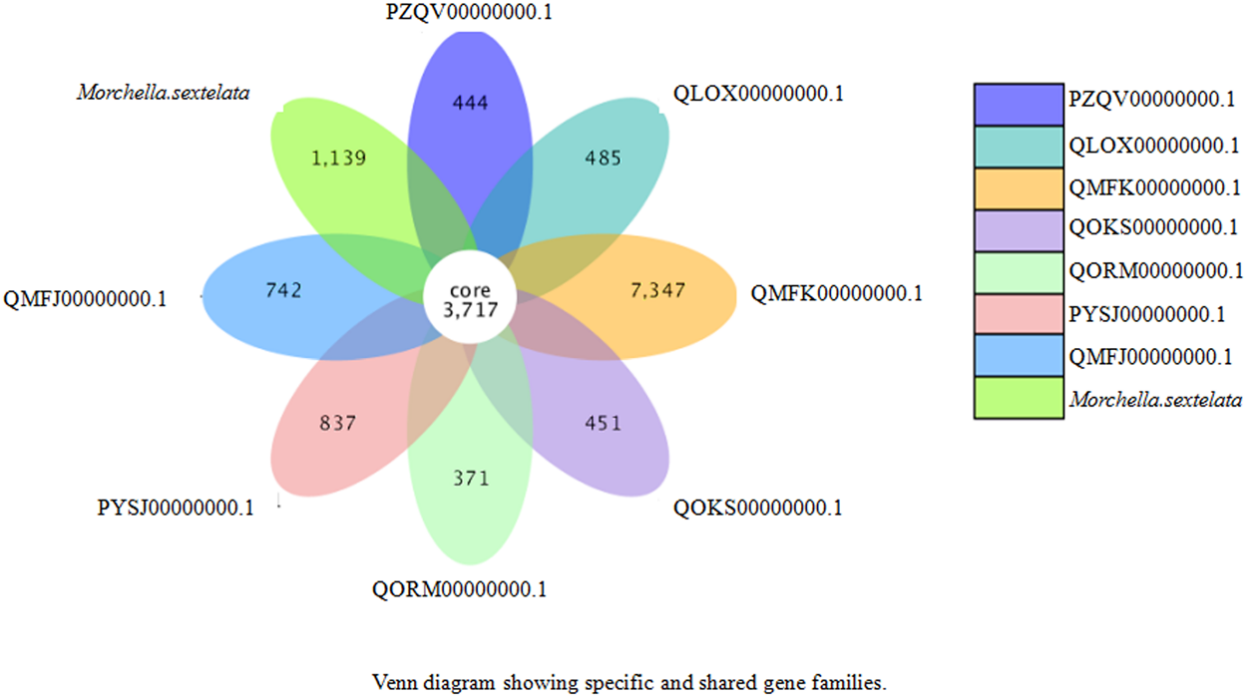
PZQV00000000.1 represents *Morchella conica* strain CCBAS932, QLOX00000000.1 and PYSJ00000000.1 represent *Morchella septimelata* strain MG91, QMFK00000000.1 represents *Morchella eximia* MG90, QOKS00000000.1 represents *Morchella importuna* M04M26, QORM00000000.1 represents *Morchella importuna* M04M24, and QMFJ00000000.1 represents *Morchella septimelata* MG113. *Morchella sextelata* is the strain studied in this work. A total of 3,717 genes written in the center indicate the gene number shared in these different *Morchella* species (or strains), while the numbers described in the diagram show the genes specifically expressed in each *Morchella* species (or strain).

Then, the shared and specific genes in different species of *Morchella* were analyzed as shown in Fig. 7. The results showed that *M. eximia* exhibited the highest specific gene families with 7,347, and *M. sextelata* also showed 1,139 specific gene families, which is much higher than the number of specific gene families in other species, ranging from 371 to 837. On the other hand, the number of shared gene families was 3,717.

**Figure 8:**
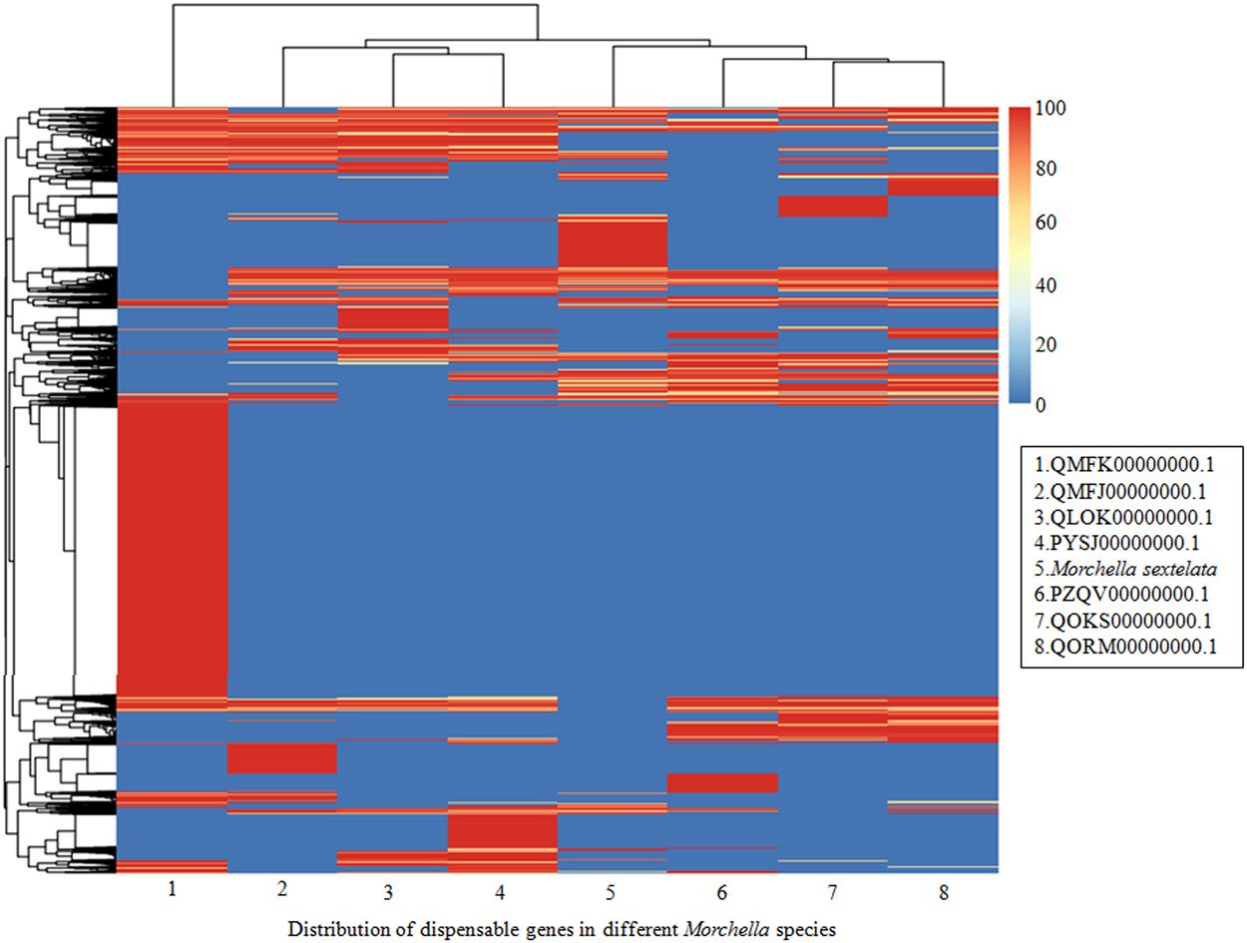
Left: the clustering tree of dispensable genes. Top: the clustering tree of samples. Middle: the expression level of dispensable genes in *Morchella* species (or strains) with different identity values, which are described with different colors, as shown on the upper right.

Furthermore, the dispensable genes, which are all genes except the core genes, in each strain were analyzed, and a heatmap is shown in Fig. 8. Consistent with the results in the Venn diagram, *M. eximia* (QMFK00000000.1) showed a significant difference compared to other *Morchella*. Additionally, a large number of specific genes were found in *M. sextelata*. For example, the gene A8276 specifically expressed in *M. sextelata* was predicted to be involved in bladder cancer, thyroid cancer, and acute myeloid leukemia. Interestingly, the gene A5703 was also functionally related to bladder cancer, thyroid cancer, and acute myeloid leukemia in these *Morchella* species.

In-depth analysis revealed that the shared genes annotated to encode PLs were A5192, A3285, A5387, A5018, A5042, and A0650 in these *Morchella* species. Comparatively, *M. sextelata* was found to have 14 specific genes encoding PLs, for example, A2625, A4964, and A7924 (Table S5), suggesting some unique biological features of *M. sextelata*. PLs play important roles in the metabolism of polysaccharides, which have been reported to have antitumor activity^4^. This indicates that comparative analysis can provide important data for conducting extensive studies on the biological function of *M. sextelata* and other *Morchella* species. (Supplementary data, dispensable matrix).

## Discussion

The genome sequencing in this study provides the whole-genome sequence of *M. sextelata* and its related gene annotation for the first time. This study may provide important data for evaluating *Morchella* species, improving culture techniques by mediating mating behavior, and discovering biologically active compounds. It is important not only for meeting the increasing demand for *M. sextelata* but also for further research on *M. sextelata*.

The comparative genomic analysis of *M. sextelata* and other *Morchella* species, *M. conica*, *M. importuna*, *M. septimelata*, and *M. eximia*, was carried out in this study. To provide additional useful information, the gene annotation file generated in this study was also uploaded and may provide useful data for further research on the differences between various *Morchella* species and their biological functions in the future.

The biological roles of *Morchella* have been reported in recent years. The potential neuritogenic activity of the aqueous extract from *M. importuna*^11^, immunomodulatory activity of the exopolysaccharides extracted from *M. conica*^12^, and antitumor activity of polysaccharides isolated from *M. esculenta* were determined^4^. In addition, a polysaccharide from *M. conica* was found to inhibit H_2_O_2_-induced oxidative stress in human embryonic kidney 293 T cells^13^. In addition to these, many other biological functions of the compounds obtained from different *Morchella* have been identified, suggesting the great value of further studies of *Morchella*.

## Materials and Methods

### *M. sextelata* sample

The self-mated *M. sextelata* spores were cultured in sandy soil in a plastic greenhouse with controlled light, temperature and humidity in Shagedu town located at the eastern end of the Ordos Plateau in Inner Mongolia, China. The *M. sextelata* isolate we cultured usually had darker caps and larger body sizes. The average size was approximately 15 cm high. It is typically planted from October to November and is picked up from April to May in the next year. Since *Morchella* grows at low temperatures, the weather of Inner Mongolia is very suitable.

### DNA extraction and quality test

1. A fresh *Morchella* sample was collected and ground completely in a mortar and pestle precooled with liquid nitrogen.
2. The sample was then transferred into a 50-ml tube containing 20 ml GP1 and 500 μl mercaptoethanol and mixed thoroughly by shaking in a 55 °C water bath. The sample was centrifuged at 12,500 rpm for 8 min (GP1 buffer: RK116-02).
3. The supernatant was collected, and saturated phenol/chloroform/isoamyl alcohol (25:24:1) was added. Then, it was centrifuged after mixing at 12,500 rpm for 8 min.
4. The supernatant was collected and mixed with chloroform/isoamyl alcohol. Then, the mixture was centrifuged at 12,500 rpm for 8 min after vortexing.
5. The supernatant was collected again in a 50-ml new tube, and isopropanol was added and mixed well. The sample was placed at −20 °C to allow precipitation for a few minutes. Then, the mixture was centrifuged (12,500 rpm, 8 min) and the supernatant was removed.
6. The pellet was washed twice with 5 ml 75% alcohol. The supernatant was removed carefully, and the pellet was dried.
7. A total of 100 μl ddH_2_O was added to dissolve the DNA sample. Then, 2 μl RNase was added to digest RNA, and the sample was then shaken several times and incubated at 37 °C for 15 min.
8. Finally, the DNA sample was purified with the PowerClean Pro DNA.
9. Clean-Up Kit (No. 12997-50), and 154 μl of DNA sample with a concentration of 107.60 ng/μl was obtained. A total of 16.570 μg DNA was prepared for further analysis.

To ensure that the harvested DNA sample was suitable for further analysis and that there was no contamination, the quality of the harvested DNA was examined by agarose gel electrophoresis.

### Genome sequencing and assembly via a second-generation system

First, the DNA was randomly cut into 350 bp fragments by Covaris ultrasonic processor. The fragments were modified with end-repairing and the addition of A tail and sequencing adaptors and were purified and PCR amplified to generate a 350 bp long fragment library. Next, the library was initially quantified by Qubit 2.0, and the insert size of fragments was tested by Agilent 2100. Then, the effective concentration of the library was examined by q-PCR to ensure that the quality of the 350 bp library was fit for further sequencing analysis. Subsequently, the genome of the *Morchella* sample in the 350 bp libraries was sequenced by the second-generation Illumina PE150 system. The generated reads were filtered by several steps to obtain the clean reads, which were then analyzed through K-mer (K-mer = 15) to estimate the genome size.

The genome for the *M. sextelata* sample was obtained by assembling the clean data from the Illumina PE150 system by SOAP de novo (version 2.04)^14^, SPAde^15^ and ABySS^16^ software and subsequently integrating the genome sequence with CISA^17^ software. The initially assembled genome was optimized and supplemented by the gapclose (version 1.12) program. Consequently, the result of the assembly was statistically analyzed by scaffolds and contigs after excluding fragments <500 bp. Finally, the GC content and coverage depth were analyzed to determine whether there was sequence contamination and repeat sequences.

All of the results obtained from the Illumina PE150 system were used as the survey for the PacBio RSII third-generation sequencing and further analyses as a source of genomic information.

### Genome sequencing and assembly via a third-generation system

After the survey was performed using the Illumina PE150 system, another part of the DNA sample was cut into 20 Kb fragments by Covaris g-TUBE, and the DNA damage and ends of the DNA were repaired. Second, both ends of the fragments were joined with a barcode. Next, these fragments were purified with AM pure PB to generate 20 Kb SMRT Bell libraries. The fragments 20 Kb in length were then screened through BluePippin and purified with AMPure PB beads. Subsequently, the library was quantified by Qubit 2.0, and the insert size of fragments was examined by Agilent 2100. After that, the genome of *M. sextelata* in the 20 Kb SMRT Bell libraries was sequenced by the PacBio RSII third-generation sequencing system. The obtained raw reads were filtered to obtain the clean reads.

The clean reads were assembled using SMRT Link v5.1.0 software (https://www.pacb.com/support/software-downloads/)^18,19^ by consulting the results from the second-generation sequencing data. De novo assembly was carried out based on the results from the Illumina PE150 system since no reference genome of other *Morchella* was available. Then, the genome obtained from the PacBio RSII third-generation sequencing system was used for all of the further analyses, including the prediction of genome size, genome annotation, genome component and gene function.

Such an assembly that combines second- and third-generation sequencing data can revise and decrease the interference of abnormal GC contents, high repetition and hybridity. Therefore, it can improve the integrity and uniformity of the generated genome sequence. It is particularly applicable for complex fungi such as *Morchella*, which lack comparable genomic information, such as reference sequences, GC contents, and repetitive sequences.

Both sequencing procedures were performed using the Illumina HiSeq. 4000 sequencing platform at Beijing Novogene Bioinformatics Technology Co., Ltd.

### Genome annotations

For *M. sextelata*, by default, we performed de novo prediction using the Augustus 2.7 program^20^ to obtain the protein-coding genes.

### Genome component predictions

Genome component prediction included the prediction of the coding genes, the repetitive sequences and the ncRNA. The interspersed repetitive sequences were predicted using RepeatMasker (http://www.repeatmasker.org/)^21^. The tandem repeats were analyzed by tandem repeats finder^22^. NcRNAs include transfer RNAs (tRNAs), ribosome RNAs (rRNAs), small RNAs (sRNAs), small nuclear RNAs (snRNAs), and microRNAs (miRNAs). The tRNA genes were predicted by tRNAscan-SE^23^ (Version 1.3.1), while the rRNA genes were analyzed by rRNAmmer^24^ (Version 1.2). The genes for sRNA, snRNA and miRNA were BLAST searched against the Rfam 3 database, annotated^25^, and then confirmed by cmsearch (Version 1.1rc4).

### Gene function

We mainly used the GO^26^, KEGG^27,28^, KOG (http://www.ncbi.nlm.nih.gov/COG/), NR^29^, TCDB^30^, P450, Pfam (http://pfam.xfam.org/), and Swiss-Prot^31^ databases to predict gene functions. A whole-genome BLAST search (E-value less than 1e-5, minimal alignment length percentage larger than 40%) was performed against the above databases. The secretory proteins were predicted by the Signal P database^32^ (version 4.1) and TMHMM (version 2.0c). Moreover, we analyzed the secondary metabolite gene clusters with antiSMASH^33^ (version 2.0.2). Additionally, carbohydrate-active enzymes were predicted by the CAZy^34^ database.

### Comparative genomics analysis

The core genes and specific genes of the reference genomes, *M. septimelata*, *M. eximia*, *M. importuna*, and *M. conica*, were compared to the genome of *M. sextelata*. The core genes and specific genes were analyzed by CD-HIT rapid clustering of similar proteins software with a threshold of 50% pairwise identity and 0.7 length difference cutoff in amino acid^35–37^. Then, the Venn diagram and heatmap were drawn to show their relationships and specificities among the samples.

## Supplementary information

The whole-genome sequence analysis of Morchella sextelata

## Data Availability

The assembled genome sequence of *M. sextelata* has been provided to NCBI with the SRA project ID PRJNA560199, and all raw data are available.
