# Supplementary material for: The whole-genome sequence analysis of Morchella sextelata
Source: Sci Rep. 2019 Oct 25;9:15376. doi: 10.1038/s41598-019-51831-4 (PMC6814724; doi:10.1038/s41598-019-51831-4)
Supplement: Supplementary file 1 — The whole-genome sequence analysis of Morchella sextelata [file 41598_2019_51831_MOESM1_ESM.pdf]

Supplementary Information

The whole-genome sequence analysis of *Morchella sextelata*

Mei-HAN[1], Qingshan-WANG[1], Baiyintala[2], Wuhanqimuge[3*]

[1] Inner Mongolia ZhongXing Agriculture and Animal Husbandry Development Co., Ltd. Ulanqab, China

[2] Inner Mongolia Mang Lai Food Co., Ltd. Hohhot, China

[3] Innovative Mongolian Pharmaceutical preparations Laboratory of Inner Mongolia, Inner Mongolia International Mongolian Hospital, Hohhot, China

[*:] Correspondence and requests for materials should be addressed to Wuhanqimuge (email: gege_403@163.com)

Supplementary Figures

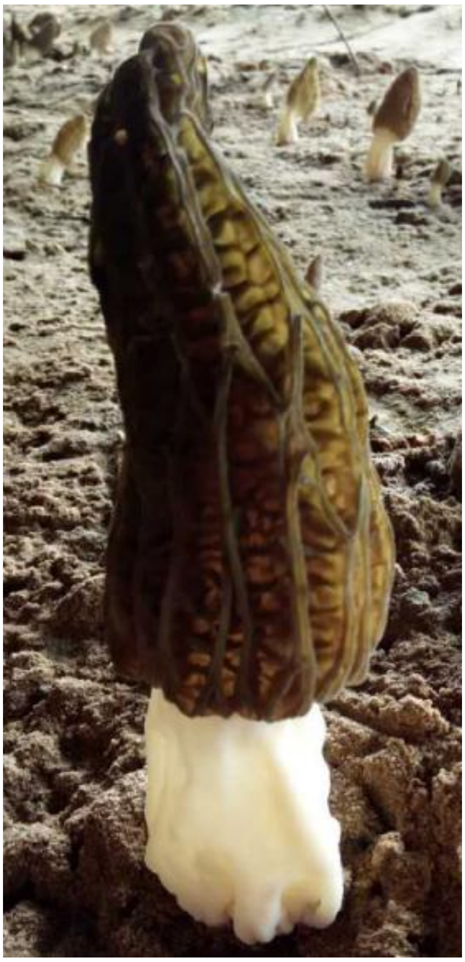

Supplementary Figure 1. ***Morchella sextelata*** **cultured in Inner Mongolia.**

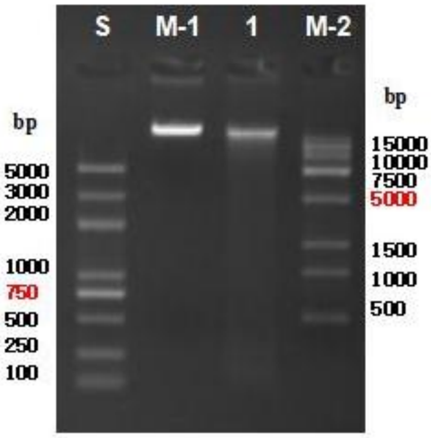

Supplementary Figure 2. Agrose gel electrophoresis of DNA from *Morchella sextelata*. *S: standard; M-1: marker 1, Trans 2k plus; 1: sample DNA; M-2: marker 2,

Trans 15k plus.

Supplementary Table 1. The summary of K-mer analysis

Sample ID	K-mer	K-mer Number	K-mer Depth	Genome Size(Mb)	Revised Size(Mb)	Heterozygous Rate (%)	Repeat Rate (%)
M.sextelata	15	1,662,360,402	27.36	60.75	58.77	0.10	36.09

Supplementary Table 2. The summary of clean data

Sample ID	Mean Concordance	Number of Reads	Number of Bases (bp)	Mean Read Length (bp)	N50 Read Length (bp)
M.sextelata	0.84	869182	5759334362	6626	10165

Supplementary Table 3. The summary of repeat sequences

Sample ID	type	Number	Repeat Size (bp)	Total length (bp)	In Genome (%)
M.sextelata	TR	15,485	1~1,894	856,046	1.6175
M.sextelata	Minisatellite DNA	10,026	10~60	475,234	0.8979
M.sextelata	Microsatellite DNA	2,702	2~6	115,567	0.2184

Supplementary Table 4. The summary of non-coding gene prediction

Type		Number	Average Length (bp)	Total length (bp)	Gene Length/Genome (%)
tRNA		445	85	37,866	0.0715
rRNA_de	5S	49	116	5,685	
	5.8S	0	0	0	
	18S	3	1,798	5,394	0.0444
	28S	3	4,134	12,402	
rRNA_ho	5S				
	5.8S				
	18S				
	28S				
sRNA		0	0	0	0
snRNA		30	145	4,373	0.0083
miRNA		0	0	0	0

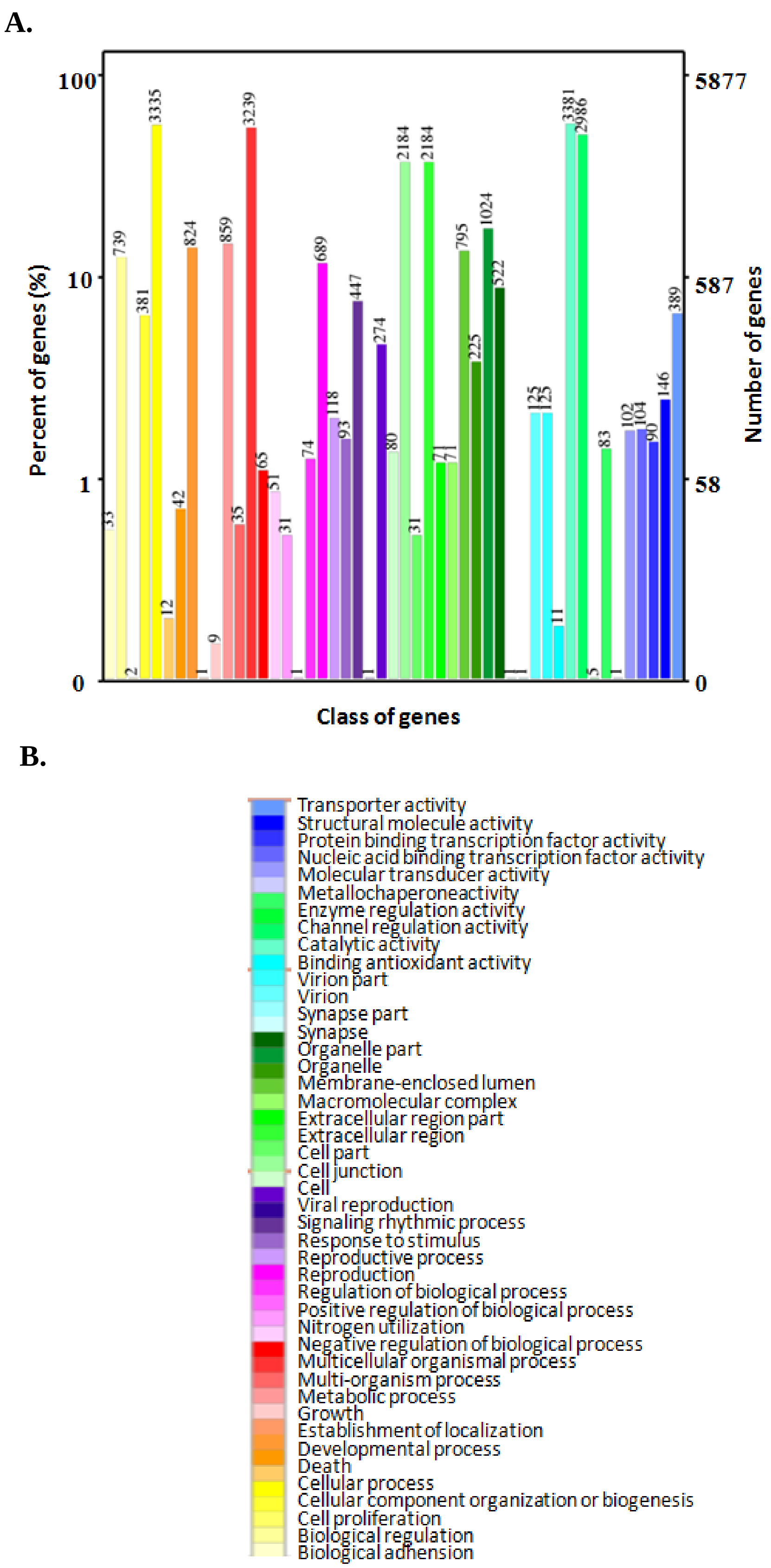

Supplementary Figure 3. The annotation of gene function for *Morchella sextelata*.

A. The genes were classified by molecular function, cellular component and biological process. **B**. The explanation of gene functions that different colors represented in A.

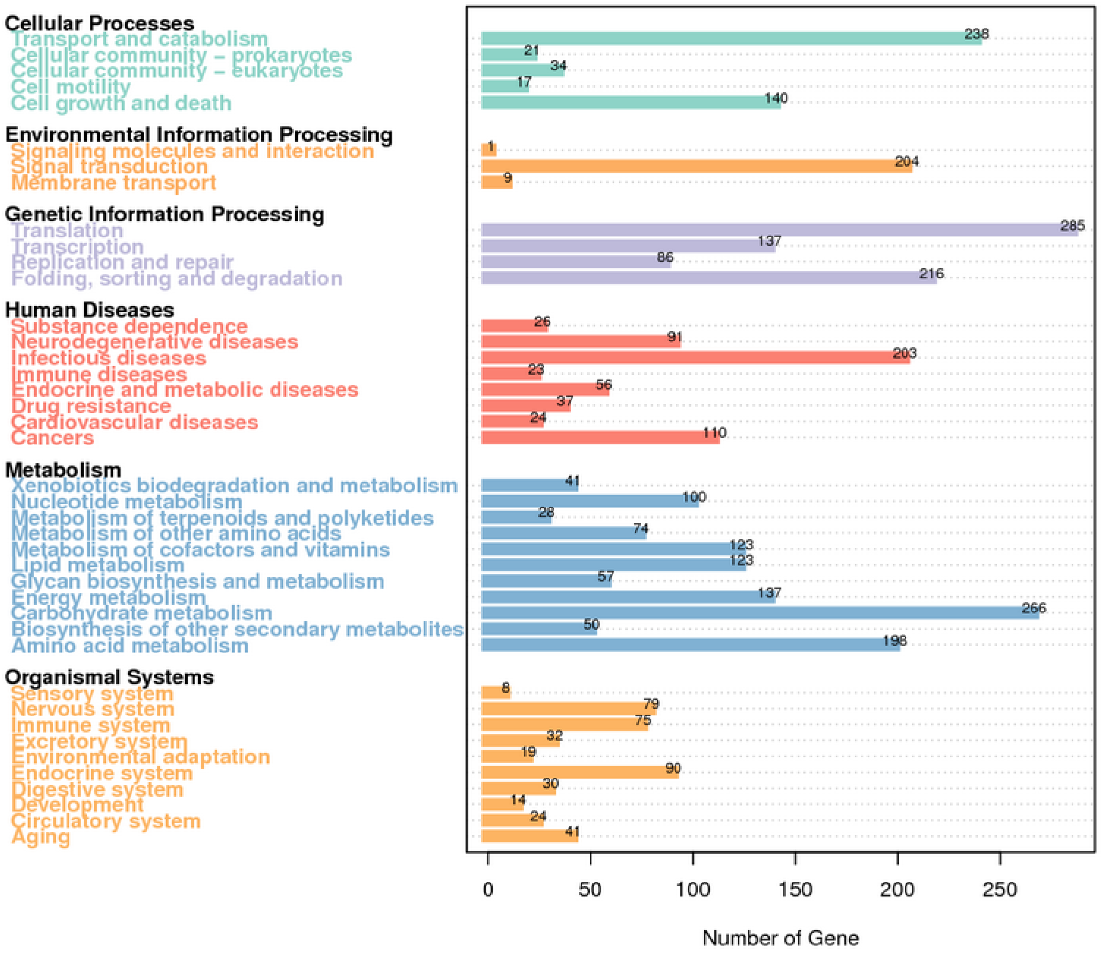

Supplementary Figure 4. KEGG pathway analysis for the genes of *Morchella sextelata.*

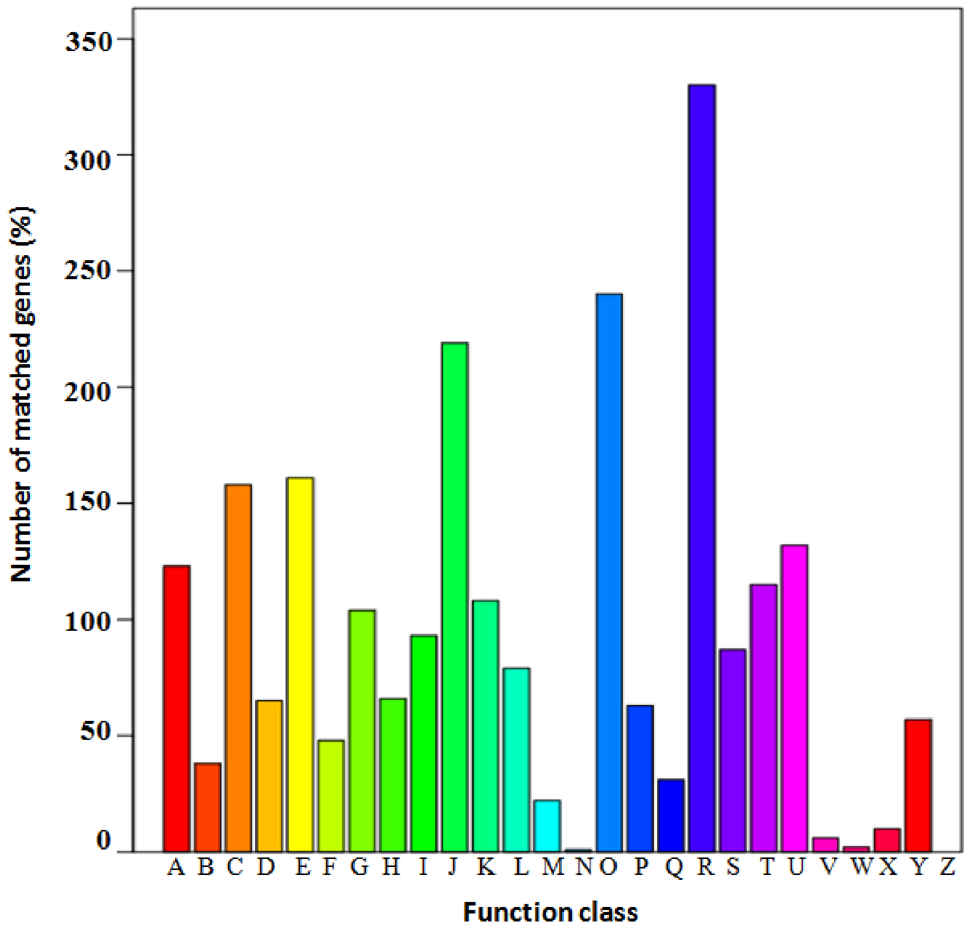

Supplementary Figure 5. The KOG function classification of the genes of *Morchella sextelata*.

A:RNA processing and modification (123), B:Chromatin structure and dynamics (38), C:Energy production and conversion (158), D:cell cycle control, cell division, chromosome partitioning (65), E:Amino acid transport and metabolism (161), F:Nucleotide transport and metabolism (48), G:Carbohydrate transport and metabolism (104), H:Coenzyme transport and metabolism (66), I:Lipid transport and metabolism (93), J:Translation, ribosomal structure and biogenesis (219), K: Transcription (108), L: Replication, recombination and repair (79), M: Cell wall/membrane /envelope biogenesis (22), N: Cell motility (1), O: posttranslational modification, protein turnover, chaperones (240), P:Inorganic ion transport and metabolism (63), Q:Secondary metabolites biosynthesis, transport and catabolism (31) R: General function prediction only (330), S: Function unknown (87), T: Signal transduction mechanisms (115), U: Intracellular trafficking, secretion and vascular transport (132), V: Defense mechanism (6), W: Extracellular structure (2), Y: Nuclear structure (10), Z: Cytoskeleton (57).

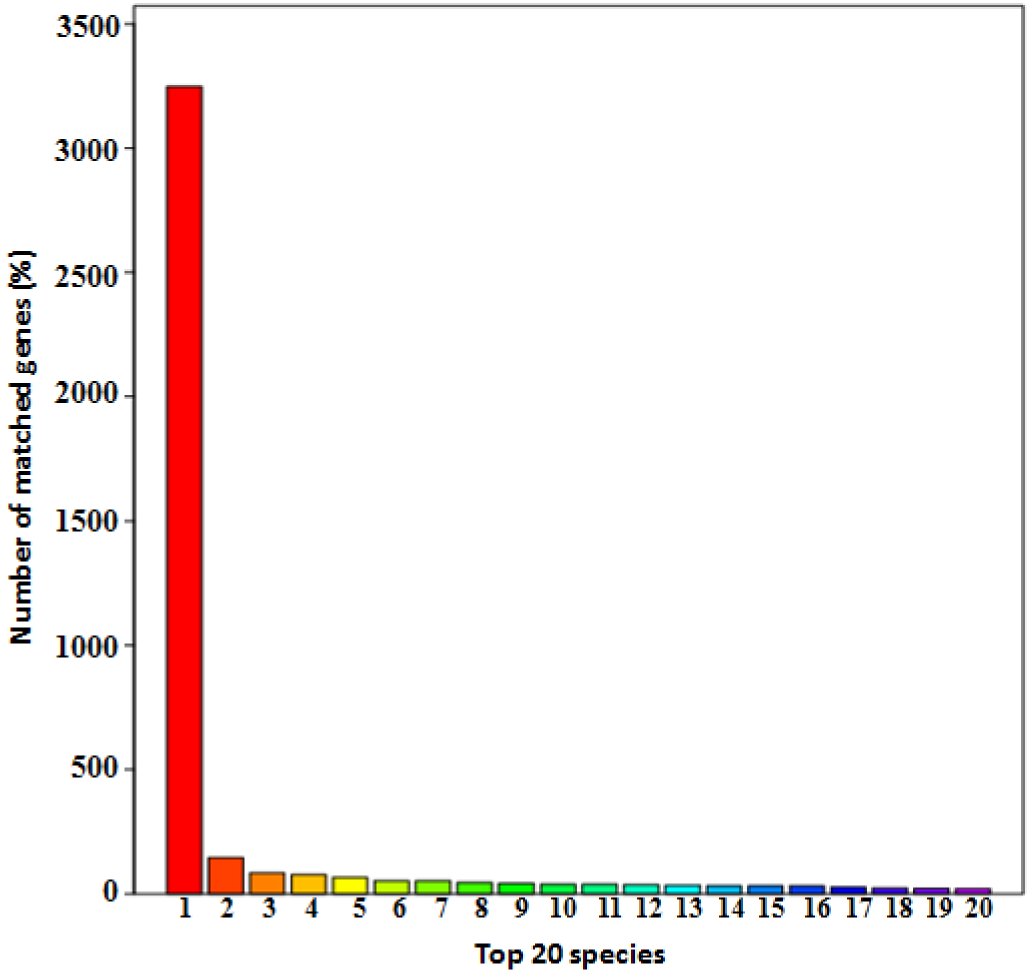

Supplementary Figure 6. The species annotation of the genes of *Morchella sextelata* in NR database. 1:Tuber melanosporum (3247), 2:Podospora anserina (146), 3.Pseudogymnoascus pannorum (84), 4: Arthrobotrys oligospora (78), 5:Conlosporium apollinis (66), 6:Claviceps purpurea (52), 7:Dactylellina haptotyla (52), 8:Neofusicoccum parvum (45), 9:Rhizoctonia solani (41), 10:Fusarium oxysporum (38), 11:Penicillium digitatum (38), 12:Glarea lozoyensis (37), 13:Macrophomina phaseolina (34), 14:Endocarpon pusillum (32), 15:Botrytis cinerea (32), 16:Cryptococcus neoformans (31), 17: Trichosporon asahii (26), 18:Drechslerella (23), 19:Trametes cinnabarina (21), 20:Ajellomyces capsulatus (20).

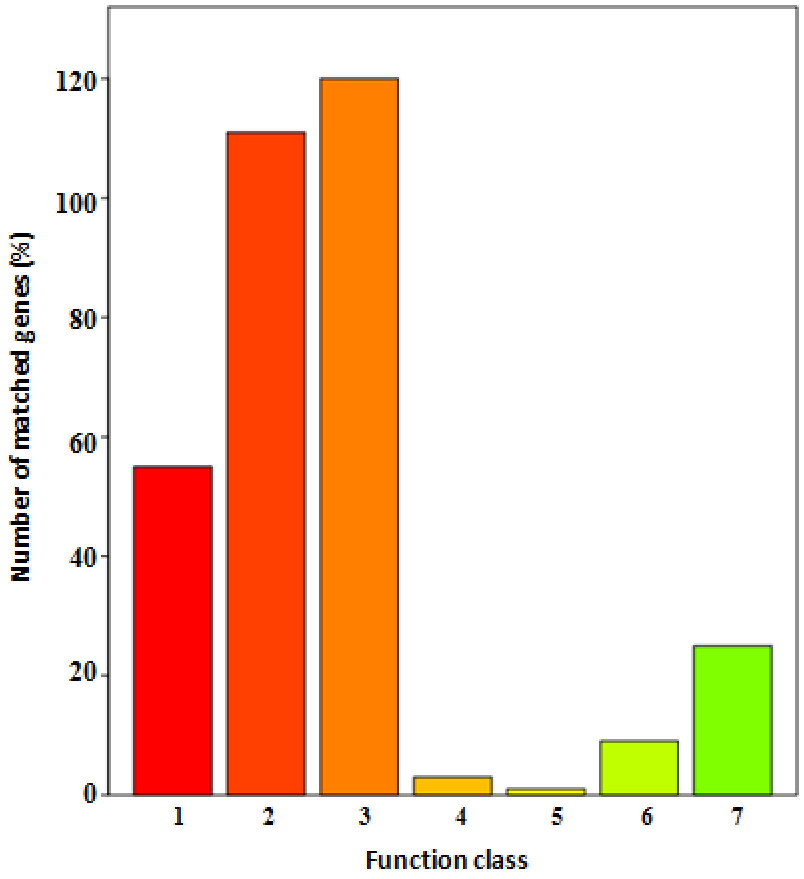

Supplementary Figure 7. The TCDB function annotation for the genes of *Morchella sextelata*. 1:Channels/Pores(55), 2:Electrochemical Potential-driven Transporters(111), 3:Primary Active Transporters(120), 4:Group Translocators(3), 5:Transmembrane Electron Carriers(1), 6:Accessory Factors Involved in Transport(9), 7:Incompletely Characterized Transport Systems(25).

Supplementary Table 5. The summary of Polysaccharide Lyases (PLs) coding genes in *Morchella sextelata*.

Type	Genes
PL1	A5018,A5042,A7924,A8087,A8860,A9147,A9148,A9370,A0650,A4245
PL3	A2625,A3008,A5192,A2262
PL4	A3285,A4964,A5387,A0273,A4082,A4822
